# Incubating parents serve as visual cues to predators in Kentish plover *(Charadrius alexandrinus)*

**DOI:** 10.1371/journal.pone.0236489

**Published:** 2020-07-29

**Authors:** Noémie Engel, Zsolt Végvári, Romy Rice, Vojtěch Kubelka, Tamás Székely

**Affiliations:** 1 Department of Evolutionary Zoology and Human Biology, University of Debrecen, Debrecen, Hungary; 2 Department of Biology and Biochemistry, Milner Centre for Evolution, University of Bath, Bath, United Kingdom; 3 Centre for Ecological Research, Danube Research Institute, Budapest, Hungary; 4 Senckenberg German Entomological Institute, Müncheberg, Germany; 5 Department of Biodiversity Research, Global Change Research Institute, Czech Academy of Sciences, Prague, Czech Republic; 6 Department of Animal and Plant Sciences, University of Sheffield, Sheffield, United Kingdom; 7 Maio Biodiversity Foundation, Maio, Republic of Cape Verde; Phillip Island Nature Parks, AUSTRALIA

## Abstract

Ground-nesting birds face many challenges to reproduce successfully, with nest predation being the main cause of reproductive failure. Visual predators such as corvids and egg-eating raptors, are among the most common causes of nest failure; thus, parental strategies that reduce the risk of visual nest predation should be favored by selection. To date, most research has focused on egg crypsis without considering adult crypsis, although in natural circumstances the eggs are covered by an incubating parent most of the time. Here we use a ground-nesting shorebird, the Kentish plover (*Charadrius alexandrinus*) as model species to experimentally test whether decoy parents influence nest predation. Using artificial nests with a male decoy, a female decoy or no decoy, we found that the presence of a decoy increased nest predation (N = 107 nests, p < 0.001). However, no difference was found in predation rates between nests with a male versus female decoy (*p* > 0.05). Additionally, we found that nests in densely vegetated habitats experienced higher survival compared to nests placed in sparsely vegetated habitats. Nest camera images, predator tracks and marks left on eggs identified the brown-necked raven (*Corvus ruficollis*) as the main visual nest predator. Our study suggests that the presence of incubating parents may enhance nest detectability to visual predators. However, parents may reduce the predation risk by placing a nest in sites where they are covered by vegetation. Our findings highlight the importance of nest site selection not only regarding egg crypsis but also considering incubating adult camouflage.

## Introduction

Predation is one of the main causes of reproductive failure in most ground-nesting birds [1, 2], and has significantly increased over the past 70 years [2, 3]. While predation plays an important role in ecosystems by controlling the growth of prey populations, it can also affect entire bird communities and lead to population declines by influencing indicators of productivity such as clutch size, hatching and fledging success or the number of broods [4–6]. Increased nest and chick predation rates disrupt the reproductive output of wild bird populations and unless compensated by high adult survival, this can alter population dynamics [7, 8]. Extreme rates of predation can lead to population declines and in exceptional situations drive populations to extinction [3, 8–10]. Because every bird is exposed to the risk of predation, predation represents a driving evolutionary force in shaping life-history traits such as nest construction, clutch site or anti-predatory tactics [11–13]. Thus, studying the factors driving nest predation and its consequences is not only an important contribution to evolutionary biology but also for species conservation [2, 3].

Ground-nesting birds including shorebirds are particularly well-suited to study nest predation because they build simple open nests on the ground that are susceptible to predators. They exhibit an impressive diversity of morphological and behavioral anti-predatory strategies during breeding [14–16]. Nest placement and visibility are obvious strategies to reduce the risk of predation. Whilst most research on nest predation has focused on the visibility of the eggs as a method to avoid detection [8, 17, 18], less attention has been paid to adult crypsis [19]. For example, recent work demonstrated that plumage coloration plays a role in nest predation in red-capped plovers (*Charadrius ruficapillus*) with more colorful males posing a higher risk for nest predation [20]. The latter result is consistent with arguments that concealed plumage of the incubating parent(s) is used to evade predators searching for nests [15]. Camouflage of eggs and adults as well as the habitat where nests are placed are important factors in avoiding predation. Predation rates may differ between different habitats because different habitats attract different predators and because of different compositions of vegetation and food availability between habitats [21–23]. Thus, all these factors influence parental nest site selection in order to avoid detection by predators [24].

To counteract parental anti-predator strategies, predators use different tactics to locate food items i.e. visual versus olfactory cues. Visually oriented predators locate their prey by walking, by scanning of large areas from elevated vantage points or coursing back and forth in the air and may use the parents’ presence to find nests [19, 25–27]. Thus, parental strategies to conceal nests and escape predation versus predator tactics for locating the nest are one of the evolutionary arms races with implications for population biology and biodiversity conservation [11, 15, 28].

Here, we investigate the potential impact of the parental presence on nest survival using bird decoys at artificial nests. Although this approach is intuitively appealing for studies of nest predation, we are aware of only three studies that investigated the impact of bird decoys on nest survival [19, 29–31]. To investigate the potential impact of parent birds on nest survival, we use the Kentish plover (*Charadrius alexandrinus*), a small widespread shorebird, as a field model system. Kentish plovers are small ground-nesting shorebirds that have been used as an ecological model species of breeding system evolution [32, 33]. Using artificial nests composed of plasticine eggs and bird decoys mimicking male and female Kentish plovers, we investigate whether the presence of incubating birds makes nests conspicuous for potential predators. Specifically, we investigate whether (i) incubating parents serve as visual cues used by predators; and whether predation rates differ (ii) between male and female parents, and/or (iii) between nesting habitats.

## Methods

### Study site

Fieldwork was carried out between 4^th^ September and 2^nd^ November 2017, the main breeding period, on the island of Maio, Cape Verde (15° 13’N, 23° 10’W). The study area Salinas do Porto Inglês is a protected Ramsar Site harboring important biodiversity and largest wetland in Maio of 535 ha [34, 35]. Approximately 100–200 breeding pairs nest in that area, and they produce between 50 and 100 clutches each breeding season [36]. The main breeding period is from early September until late November [37]. The predation pressures on the local Kentish plover population are high with low hatching success in recent years. Most egg losses are attributed to predation by brown-necked ravens (*Corvus ruficollis*) and ghost crabs *Ocypode* spp. [38]. The current study was conducted in three distinct habitats (see S1 Appendix): The Grassland was composed mainly of short grass with a small number of shrubs on a sandy substrate; the Saltmarsh was a long sandy stretch connected to the beach and colonized by an introduced halophyte plant, *Sesuvium portulacastrum*. The Semidesert constituted of mud, volcanic rocks and acacia trees. The Grassland and Semidesert were located on the north side of the Salinas Porto Inglês, the Semidesert was located on the south side of the Salinas.

### Design of artificial clutches and decoys

The experiment was carried out using artificial nest scrapes using decoy eggs and decoy adults (see below) in two sets of trials. Therefore, the artificial nests have no perceivable impact on the breeding Kentish plover population and no ethics approval was necessary. No methods of anesthesia, euthanasia or any kind of animal sacrifice were used in our study. For artificial nests, clay eggs were used for the first trial. However, as it was not possible to see tooth or beak marks of predators left on the clay, in the second trial plasticine was used to create eggs because it remained soft and marks of predators were recognizable. The protocol to prepare the eggs was the same for both materials: 11 ± 1 [SD] g of cream-colored clay/plasticine was molded into the basic shape of an egg, then rolled in small fragments of dark brown clay or plasticine and speckled with brown acrylic paint to create a realistic look providing a pattern to mimic plover eggs. The artificial eggs were 31 ± 2 [SD] mm in length and 23 ± 1.5 [SD] mm in width. A string of 50 cm ± 1 [SD] cm was attached to each egg, and the other end of the string was pegged into the ground to stop predators from removing the eggs from the nest. In total, 90 clay eggs and 126 plasticine eggs were used in Trial 1 and Trial 2, respectively.

The shape of an incubating plover was formed using scrunched-up newspaper and then covered in tin foil. As the decoys aimed to replicate incubating parents, only the main body, head, tail and bill were formed whilst omitting the legs. Then instant papier-maché was mixed with water resulting in a white paste, which was applied over the basic plover shape and left to dry for 2–3 days. In total, 30 male and 30 female decoys were produced, all measuring between 16 ± 1 cm from head to tail with a bill of 10 ± 1 mm. After drying, the models were painted with acrylic paints. Since male and female Kentish plovers have different plumage [18], we painted male decoys with darker horizontal head bars, ear coverts and uncompleted breast bars around the neck, whereas in female decoys these body parts were light brown. The main body in males was of creamy gray whereas in females it was rufous brown.

### Experimental design

In Trial 1 and Trial 2, 45 and 63 artificial nests respectively (each with 2 eggs) were evenly distributed in the three habitats. Whilst maintaining 30 meters between each nest, the nests were placed in a random spatial design to prevent predators from recognizing any patterns. All nests were placed in the morning between 6.30am and 12.30pm. Inconspicuous marks such as rocks, plants and/or rubbish found in the Salinas were used as local landmarks to relocate the nests. Nest sites were chosen to mimic natural plover nests. Small nest scrapes were excavated, and two artificial eggs were placed into each nest scrape. Using a metal stick, the string attached to the eggs was pushed into the soil beneath the eggs. The nest was lined with some nest material such as pebbles, shells or dried Sesuvium plants. The three treatments consisted of a male decoy, a female decoy placed on top of the eggs or no decoy at the nest–the latter serving as control (see S2 Appendix). Prior to the deployment of the eggs, the different treatments were allocated randomly to each nest using a random number generator. For each nest, habitat, UTM coordinates, date, time, treatment type and mark were recorded. Photos of all nests were taken with a gray card placed nearby and a label stating the nest number, date, time, treatment and habitat.

### Nest monitoring

Each trial lasted 4 days including the day of nest deployment. All nests were checked daily by NE aided by JW, MG or RR in the mornings between 6.30am and 11.30am, and in the evenings between 4.30pm and 7.00pm (local time). Nests were only approached when they seemed predated and handling time was minimized to reduce potential disturbance.

Nest fate was recorded as either predated or survived (see Table A in S3 Appendix). Nests were defined predated when the eggs exhibited predator tooth (or beak) marks, or when they appeared to have been moved away from the nest scrape. An event was also considered predation when the decoys were found away from the original nest site or showed obvious signs of predation i.e. ripped apart, deep incisions or head missing. Tracks around the nest and all changes of the nest i.e. the eggs or the decoys were recorded. The eggs and the decoys of predated nests were collected and further examined. A photo of every predated nest was taken including the nest number, date, time and habitat. If the nests appeared unchanged or unharmed at the end of the experiment, the nest fate was recorded as survived. In the prospect of capturing predation events, five Bushnell Trophy nest cameras were randomly placed one to two meters away from nests. At the end of the experiment, all remaining eggs and decoys were collected. No eggs or decoys were reused in this study.

### Statistical analyses

One nest was excluded from all analyses due to an investigators’ error, thus from here onwards, we consider 107 nests in total. A map showing the three distinct habitats of the study area was created (see S1 Appendix). Two further maps showing the artificial nest distribution were computed using GPS coordinates of nests—one map each for Trial 1 and Trial 2 (see S1 Appendix). All maps were created in QGIS 3.6.1. [39] using vector tiles from MapTiles [40]. The median survival time of artificial nests (in hours) with their upper and lower quartiles was calculated in R. From the evidence found at the nests, the type of suspected predators was also summarized (see Table B in S3 Appendix).

To investigate nest survival, we performed a Generalized Linear Model (GLM) fitted on estimated nest survival time as a function of treatment and habitat. Estimated nest survival time entered into this model was calculated using ST = a + b/2, where ST is the hours that each nest survives, a is the time between the initial nest placement and the last time a nest was found unharmed (in hours) and b is the time between the last time a nest was found unharmed and the time of predation (in hours). We entered a log(ST+10) transformed version of ST into the GLM model. To test factor effects, we performed a chi-squared test on the GLM model’s results. To visually represent estimated survival time in relation to treatment and habitat, we created a box plot figure using ggplot2 in R using estimated survival time in hours except for those nests that survived until the end of the trials, for which we used the maximum survival [41].

In addition, to test the robustness of the GLM analysis, a multiple Cox hazard ratio analysis was carried out using the “survival” package [42, 43] in the R statistical programming environment. For this model nest predation was the terminal event, whereas nests surviving until the end of the experiment were considered censored. Survival time entered into this model was the time between the start of the experiment and either the terminal event or the end of the experiment.

We computed two Cox hazard ratio models. In the first model, treatment, habitat and trial were entered as factors to analyze which ones were associated with reduced nest survival. In this model trial did not predict nest survival, therefore, trial was not included in further analyses (see Table C in S3 Appendix for details). In the second Cox hazard ratio model, treatment and habitat were entered as factors and first-order interaction between treatment and habitat was also included in the model. Additionally, to test factor effects instead of individual factor levels, we performed a chi-squared test on the Cox hazard ratio model’s results. To give a visual representation of the data and facilitate interpretation, survival curves were produced separately for treatment and survival using the “survminer” function in the CRAN package [44] and ggplot2 [41] in R.

To analyze the effect of treatment and habitat on the type of predator (visual versus non-visual), a binomial GLM was computed excluding non-predated nests [45]. When we included interaction between treatment and habitat, the full model output included several non-significant terms. In the next step, we excluded the term with the largest p value (interaction between Treatment and Saltmarsh) to obtain the next model as part of a backward elimination process. In the final step, we were unable to remove any other factors, thus achieved the minimal model. Similarly, to analyze the effect of treatment and habitat on the timing of nest predation (daytime versus nighttime), a binomial GLM was computed also excluding non-predated nests. Similarly, no interaction between treatment and habitat was found and it was thus excluded from the model. All statistical analyses were performed using the program R.2.72 [46] and results were considered significant with p < 0.05.

## Results

### Frequency of nest predation

16 nests out of 107 nests from the two trials (17%) survived the end of the trials: 15 of these were a control nest and one was a nest with a male decoy (Table 1). The main nest predators were brown-necked ravens (*Corvus ruficollis*) and ghost crabs (*Ocypode* spp.) based on multiple cues of evidence of predation such as camera evidence, eyewitnesses and marks or tracks that predators left on the eggs, the decoys or the surroundings of the nest site (see S2 Appendix for full details). 10 confirmed predation events were by ravens: 5 recorded by nest cameras and five were recorded by the observers.

**Table 1:** Survival summary of the three treatments *Male decoy*, *Female decoy and Control* in the three distinct habitats *Grassland*, *Saltmarsh and Semidesert* for Trial 1 and Trial 2.

	Male decoy	Female decoy	Control	Total
Trial 1				
**Grassland**	39.11 (29.52–41.21, 5)	29.64 (29.11–52.39, 5)	82.23 (82.05–82.80, 5)	41.21 (29.58–67.47, 15)
**Saltmarsh**	28.50 (27.77–35.72, 5)	28.86 (28.39–38.53, 5)	28.66 (27.73–28.79, 5)	28.66 (27.75–37.12, 15)
**Semidesert**	33.17 (33.09–33.26, 5)	33.15 (33.01–33.37, 5)	78.95 (33.55–79.15, 5)	33.34 (33.12–33.52, 15)
	.	.	.	33.34 (29.11–41.21, 45)
**Trial 2**				
**Grassland**	40.81 (30.92–38.67, 7)	40.49 (16.17–40.64, 7)	93.26 (81.86–93.42, 7)	40.86 (33.16–81.80, 21)
**Saltmarsh**	39.33 (23.04–39.92, 7)	39.62 (39.08–39.88, 7)	14.81 (14.66–15.11, 7)	38.48 (15.10–39.71, 21)
**Semidesert**	25.49 (25.29–25.89, 7)	25.50 (25.47–30.53, 7)	43.51 (36.94–80.08, 6)	25.93 (25.48–36.89, 20)
	.	.	.	37.15 (25.48–40.79, 62)

Median survival hours (Lower Quartile-Upper Quartile, N) are given separately for trial one (N = 45 nests) and trial two (N = 62 nests).

### Nest survival in relation to habitat and treatment

**Fig 1:**
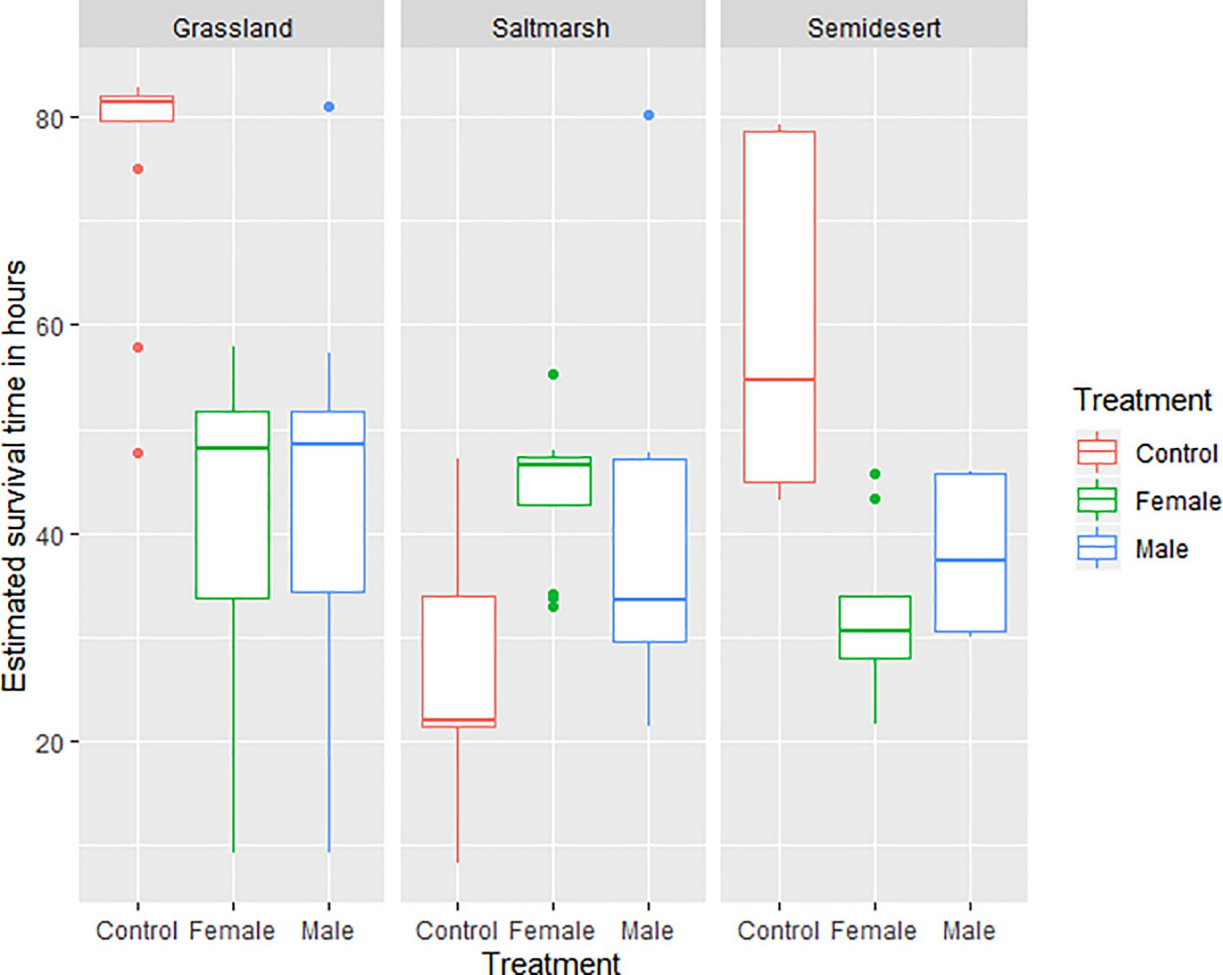
Box plots showing estimated nest survival in relation to habitat and treatment. Total number of nests: N = 107.

Both habitat and treatment influenced nest survival, although a significant interaction between habitat and treatment indicated that the presence of a decoy was not uniform between habitats (GLM results, Tables 2 and 3). The statistically significant interaction was due to control nests surviving better in Grassland and Semidesert habitats than nests with decoys, although in the Saltmarsh habitat, control nests had lower survival than nests with decoy (Fig 1). Predation of nests with male and female decoys was not different (LS-mean, *p* = 0.97)

**Table 2:** General Linear Model using estimated nest survival as response variable: Full model (N = 107 nests).

	Estimate	Std. Error	Sig.
**Treatment**			
Control	1	.	.
Male decoy	- 0.70	0.13	< 0.001
Female decoy	- 0.88	0.13	< 0.001
**Habitat**			
Grassland	1	.	.
Semidesert	- 0.34	0.13	0.01
Saltmarsh	- 1.12	0.13	< 0.001
**Interaction**			
Female x Semidesert	0.41	0.19	0.03
Male x Semidesert	0.21	0.19	0.26
Female x Saltmarsh	1.34	0.18	< 0.001
Male x Saltmarsh	1.03	0.18	< 0.001

**Table 3:** General Linear Model using log(x+10) transformed estimated nest survival as response variable: Factor effects shown (N = 107 nests).

Nest survival		Deviance	Df	Sig.
	Treatment	1.97	2	< 0.001
	Habitat	1.97	2	< 0.001
	Treatment x Habitat	6.38	4	< 0.001

**Fig 2:**
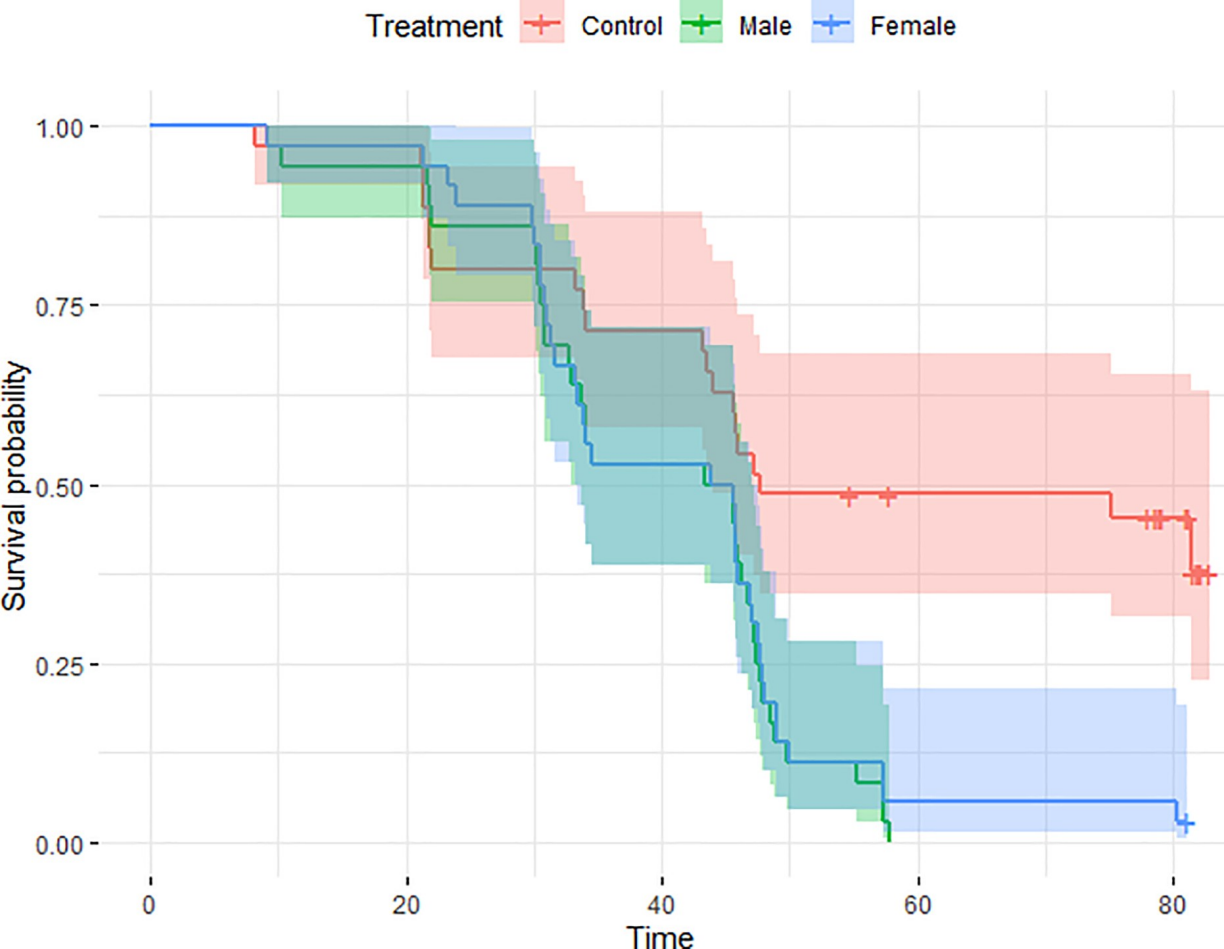
Nest survival in relation to presence of male decoy (N = 36), 95% CI (33.2–47.0), female decoy (N = 36), 95% CI (33.0–47.0) and control (N = 35), 95% CI (44.0—NA). Total number of nests: N = 107.

**Fig 3:**
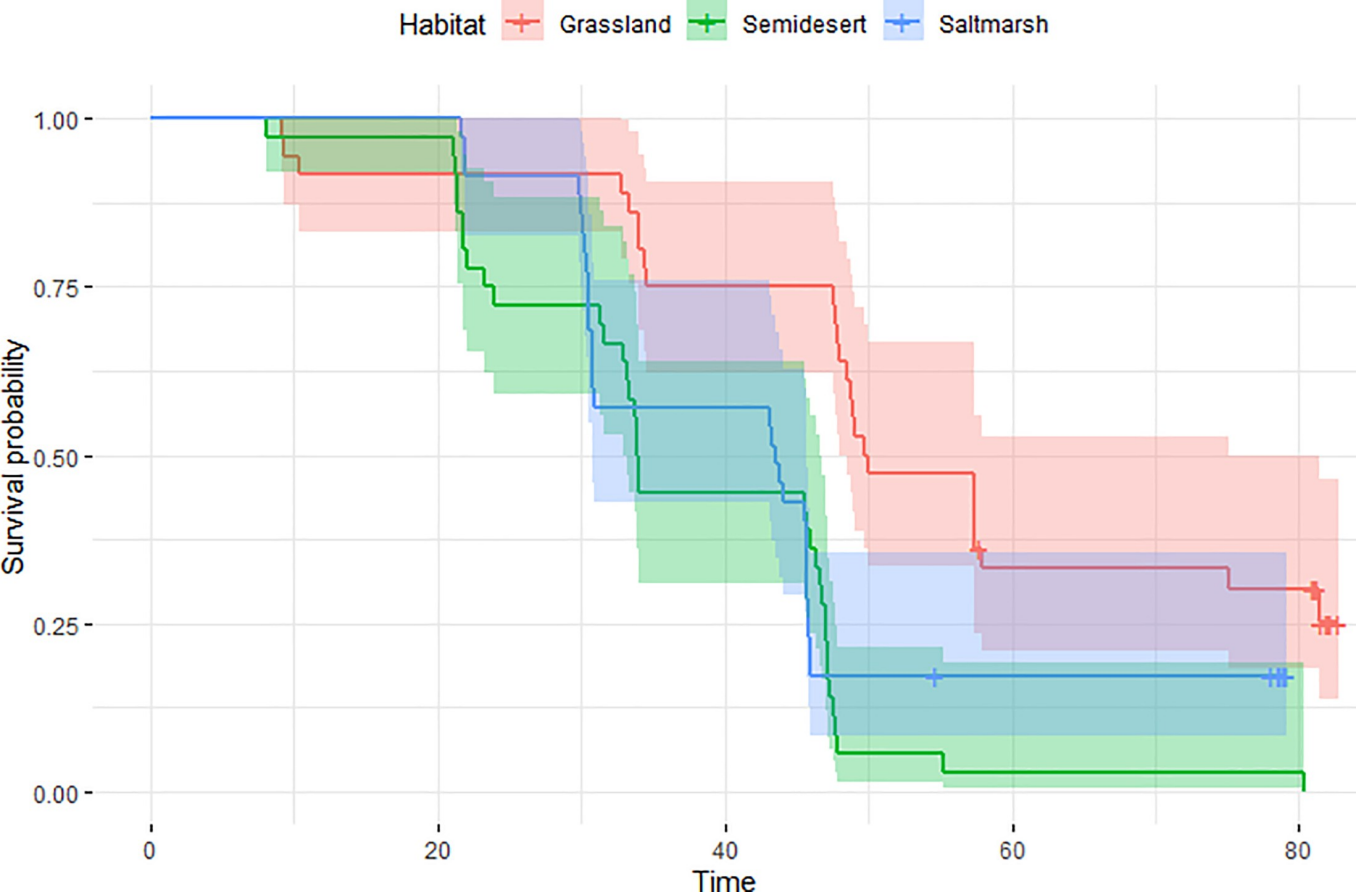
Nest survival in relation to habitats: Grassland (N = 36) 95% CI (48.0–75.1), Saltmarsh (N = 36), 95% CI (33.0–46.7) and Semidesert (N = 35), 95% CI (30.8–45.8). Total number of nests: N = 107.

These results remained consistent using survived nests as censored observations in a Cox hazard ratio model (Table 4), since habitat, treatment and interaction between habitat and treatment remained significant in these models (Figs 2 and 3, Table 5). Consistently with GLM, nest survival with male and female decoys were not different (LS-mean, *p* = 0.64).

**Table 4:** Nest survival in relation to treatment (male decoy, female decoy and control) and habitat (Grassland, Saltmarsh and Semidesert).

	HR	95% CI	Sig.
**Treatment**			
Control	1	.	.
Male decoy	12.30	2.67–56.61	0.001
Female decoy	16.03	3.49–73.57	< 0.001
**Habitat**			
Grassland	1	.	.
Semidesert	4.81	0.92–25.10	0.06
Saltmarsh	94.26	19.51–455.33	< 0.001
**Interaction**			
Female x Semidesert	1.25	0.18–7.59	0.82
Male x Semidesert	1.00	0.16–7.02	0.99
Female x Saltmarsh	0.02	0.003–0.09	< 0.001
Male x Saltmarsh	0.02	0.004–0.15	< 0.001

Cox hazard ratio model (N = 107 nests and number of events = 91, CI = confidence intervals, HR = Hazard ratio).

**Table 5:** Nest survival in relation to treatment, habitat and the interaction between treatment and habitat.

Nest survival		Chi-square	Df	Sig.
	Treatment	20.49	2	< 0.001
	Habitat	21.92	2	< 0.001
	Treatment x Habitat	46.37	4	< 0.001

Cox hazard ratio model: factor effects shown (N = 107 nests and number of events = 91, Df = degrees of freedom).

### Type of predator in relation to treatment and habitat

The type of predator (visual versus non-visual) was related to the treatment (GLM, F = 7.84, *p <* 0.01, Table 6) but not habitat (GLM, F = 2.79, *p* = 0.06, Table 6). Post-hoc Tukey-HSD tests found that nests with male (*p* < 0.01) and female (*p* < 0.01) decoys were more likely to attract visual predators compared to control nests. No difference between male and female was found in attracting visual predators (*p* = 0.72). The location of a nest did not influence the type of predators approaching nests (*p* = 0.06).

**Table 6:** Type of predators in relation to habitat and treatment (General Linear Model using visual/olfactory predator as response variable, N = 91 nests).

Type of predator		Deviance	F value	Sig.
	Treatment	111.10	7.84	< 0.001
	Habitat	100.06	2.79	0.06

### Timing of predation in relation to treatment and habitat

The timing of nest predation events was not predicted by treatment or habitat (GLM, treatment: F = 0.47, *p* = 0.62; habitat: F = 0.78, *p* = 0.46, Table 7) since nests were equally as likely to be predated during the daytime as during the nighttime.

**Table 7:** Timing of predation in relation to habitat and treatment (General Linear Model using daytime/nighttime as response variable, N = 91 nests).

Timing of predation		Deviance	F value	Sig.
	Treatment	121.85	0.47	0.62
	Habitat	122.72	0.78	0.46

## Discussion

Focusing on the role of adult crypsis and habitat structure in shorebird nest predation, our study provides four key results. First, the presence of incubating parents appears to serve as visual cue to predators in Kentish plovers. The presence of a male and female decoy significantly increased the probability of nest predation suggesting that adult crypsis plays a bigger role in visual nest predation than previously thought. Until now, it was believed that visual predators locate their food either incidentally or by delayed nest predation [19]. Our results suggest that visually oriented predators such as corvids actively search for the presence of adults incubating a nest. This is a striking result that may explain parental nest defense strategies such early silent departure from the nest when a predator approaches, a common defense behavior seen in Kentish plovers [47]. While for some species such as nightjars (*Caprimulgus* spp.), their highly camouflaged plumage combined with remaining stationary is their main protection against predators [48, 49], in other species a high concealment is associated with higher vulnerability. An experimental study on pied flycatchers (*Ficedula hypoleuca*) found that more conspicuous male black and white decoys were attacked less often by visual predators (i.e. sparrow hawks) than drab female decoys. This showed that drabness was associated with vulnerability and higher predation [15, 50]. Thus, in Kentish plovers, although their drab plumage seems to be well camouflaged against the background, they might be more vulnerable to predation and visible to predators. This might explain why Kentish plovers flush from the nest challenging predators to locate the well-camouflaged eggs. Our results support this, as only control nests (and one decoy nest) survived throughout the experiment because eggs alone are harder to find and incubating birds easier to spot.

We hypothesized that sex differences in detectability and ability to escape might influence predation rates. However, we found no difference in predation rates between the two sexes. It might be that the difference in appearance between the two sexes was too slight and in future studies, differences between sexes should be replicated more accurately. Alternatively, predators might simply not distinguish between sexes and predate nests protected by males and females equally. Additionally, we cannot rule out that brown-necked ravens perceived the decoys as novelty objects and not as incubating adults, investigating them purely out of curiosity. Novelty has been shown to influence predator-prey interactions at different steps of the predation sequence [51], thus the presence of the decoys may have altered the brown-necked raven’s usual predation mechanism. In addition, at predator approach the incubating parent would in reality leave the nest whereas during our experiment the predator had to remove the decoy to get to the eggs. In another plover species, parents rely on egg crypsis as anti-predator strategy rather than plumage crypsis, which is why they leave the nest upon predator approach [52]. The lack of this parental anti-predator behavior may explain why the decoys in our study were predated so frequently.

Second, our results show that habitat structure influences nest survival and that decoy nests survived better in the Saltmarsh habitat. This may be due to neophobia i.e. ravens looking for eggs in this area were somehow scared by the decoys. In the other two habitats, control nests survived better than nests with decoys, most likely due to higher cover by small shrubs. Predation pressures may vary in the three experimental zones in terms of type as well as the number of predators e.g. the Saltmarsh area had a vastly higher number of crab burrows (NC Engel, personal observation) than both the Grassland and the Semidesert. Furthermore, physical isolation of the Semidesert from the other two habitats by a water body may allow for predator populations to differ. This result may explain parental strategies and nest site selection of parents. They might prefer nest sites with higher nest coverage although this is evidently coupled to the trade-off of delayed approaching predator detection [47]. Taken together, these two results may explain the evolution of parental strategies. Because the interaction between treatment and habitat influences nest predation, for parents it is not only important to be well-camouflaged but also to decide what habitat to nest in to minimize the predation risk.

Third, we found that whether nests were predated by a visual predator or not, a brown-necked raven in this case, was influenced by the type of treatment a nest was undergoing and not by what habitat a nest was placed in. Most nests were predated by brown-necked ravens and ghost crabs. Brown-necked ravens are corvids, generalist omnivores foraging by mainly using visual cues. They pose a threat to natural breeding populations of numerous ground-nesting bird species that suffer from low hatching success e.g. red-capped plover (*Charadrius ruficapillus*) or greater sage-grouse (*Centrocercus urophasianus*) [53, 54] because their populations increase worldwide due to urbanization [53, 55]. Ghost crabs mainly predated artificial nests in the Saltmarsh habitat. They have highly developed senses of sight, smell and taste. However, to forage, they mainly use chemical volatile cues that they receive with chemoreceptors located on their dactyls [56, 57]. They use mainly olfactory cues to forage; thus we think the unnatural smell of plasticine might have attracted them to the nests and they were solely interested in the eggs. It is unlikely that ghost crabs were attracted to the nests by incubating parents, thus adult conspicuousness does not play a role in ghost crab predation. Evidence of both brown-necked ravens and ghost crabs together was recorded in some nests. Ravens may have investigated the decoys at dusk, leaving the eggs when they realized they were not real and at night, the nocturnal ghost crabs then investigated the eggs, so that nests were found with evidence of both predators nearby.

Fourth, our results showed that the timing of predation was not affected by the type of treatment, the habitat or the trial number, which means that nests were predated at random times.

Nest predation rates have increased globally over the past 70 years [2, 3], which is why it is important to understand how our findings relate to other shorebird species. The role of adult crypsis has mostly been overlooked in predation studies and it would be of use to know whether our results are also seen in other ground-nesting species. If so, the significance of our results goes beyond behavioral ecology and serves as an important basis of biodiversity conservation. Specifically, our findings can be used both to protect other shorebird species but in a first step to specifically protect the Kentish plover population in Maio, which is vulnerable due to increasing pressures from urbanization and also because islands count among the most endangered habitats worldwide [58]. Our results can serve as a conservation tool to design targeted anti-predator measures such as nest exclosures. These have been successfully used in numerous other shorebird species [59–62]. Such strategies may have significant short-term and/or long-term implications on the survival of nests [63]. Nevertheless, this study, while novel in its approach, also had its limitations, so that we would recommend repeating this study increasing the sample sizes, to produce more realistic decoys and to improve nest site characteristics. We would also recommend carrying out studies of factorial design to test for visual and olfactory cues to enlighten how different types of predators (visual versus olfactory) use cues conjointly to locate nests. This would uncover more about the interaction of nest site characteristics and the sensory capabilities of predators. Although artificial nest experiments provide undeniable advantages such as the control of numbers and distribution of nests by investigators, there has been a concern about the use of artificial nests among researchers. They only monitor the predation risk and not the predation rate itself because the behavior of the parent is missing, which is why testing this on real nests and parents is necessary in future studies. Results of artificial nest studies need to be interpreted carefully as they may not be consistent with ones found with real nests [64]. In our study, 15% of artificial Kentish plover nests survived, whereas during the breeding seasons of 2015, 2016 and 2018 an average of 23.5% of real nests produced vital offspring (Engel *et al*., unpublished data), suggesting a tendency towards higher numbers of nest losses in artificial nests.

In conclusion, we provide the clear evidence that visual predators such as corvids recognize the presence of incubating parents in Kentish plovers and may use this as a cue to find their food sources. The nesting habitat also plays a role in predation risks where nests placed in shrubby/grassy habitats have better chances of surviving. The main nest predators of this island population seem to be brown-necked ravens and ghost crabs, therefore targeted anti-predator strategies are needed to increase nest survival.

## Supporting information

S1 Appendix Details about the three different habitats and the artificial nest distributions for both trials in those three habitats in the Salinas do Porto Inglês, Maio. (DOC)

S2 Appendix Details of artificial nest design and the events of predation. (DOCX)

S3 Appendix Summary of nest fates, suspected predators, frequency of nest predation and the details of the full Cox hazard ratio model. (DOCX)
