# Supplementary material for: Incubating parents serve as visual cues to predators in Kentish plover (Charadrius alexandrinus)
Source: PLoS One. 2020 Jul 29;15(7):e0236489. doi: 10.1371/journal.pone.0236489 (PMC7390395; doi:10.1371/journal.pone.0236489)
Supplement: S1 Appendix — (DOC) [file pone.0236489.s001.doc]

**S1 Appendix**


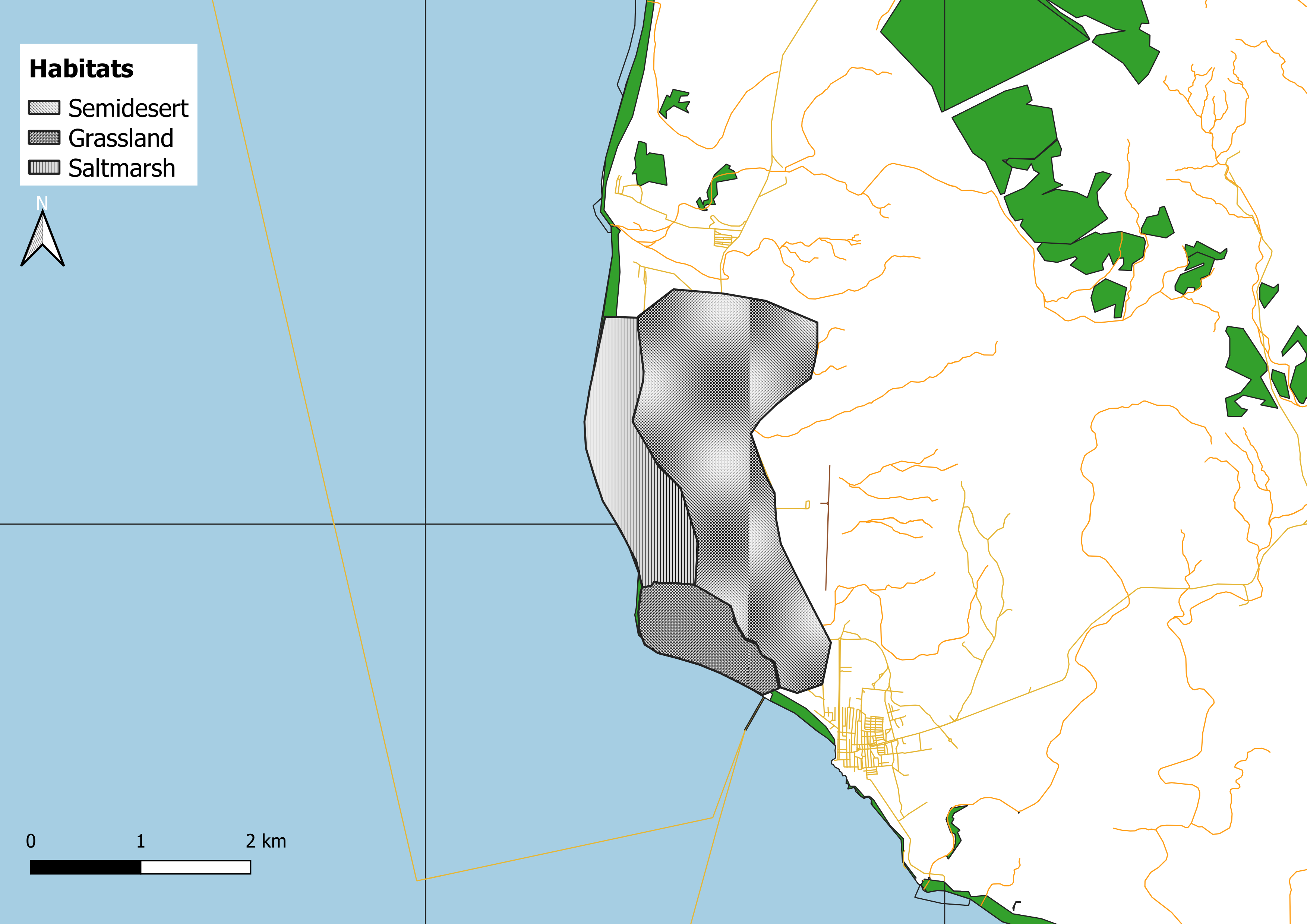


Map showing the three different habitats of the Salina do Porto Inglês in Maio, Cape Verde. The three different habitats are: Grassland, Saltmarsh and Semidesert. This map was created using purchased vector tiles of Cape Verde from © MapTiler © OpenStreetMap contributors and then manipulated in QGIS.


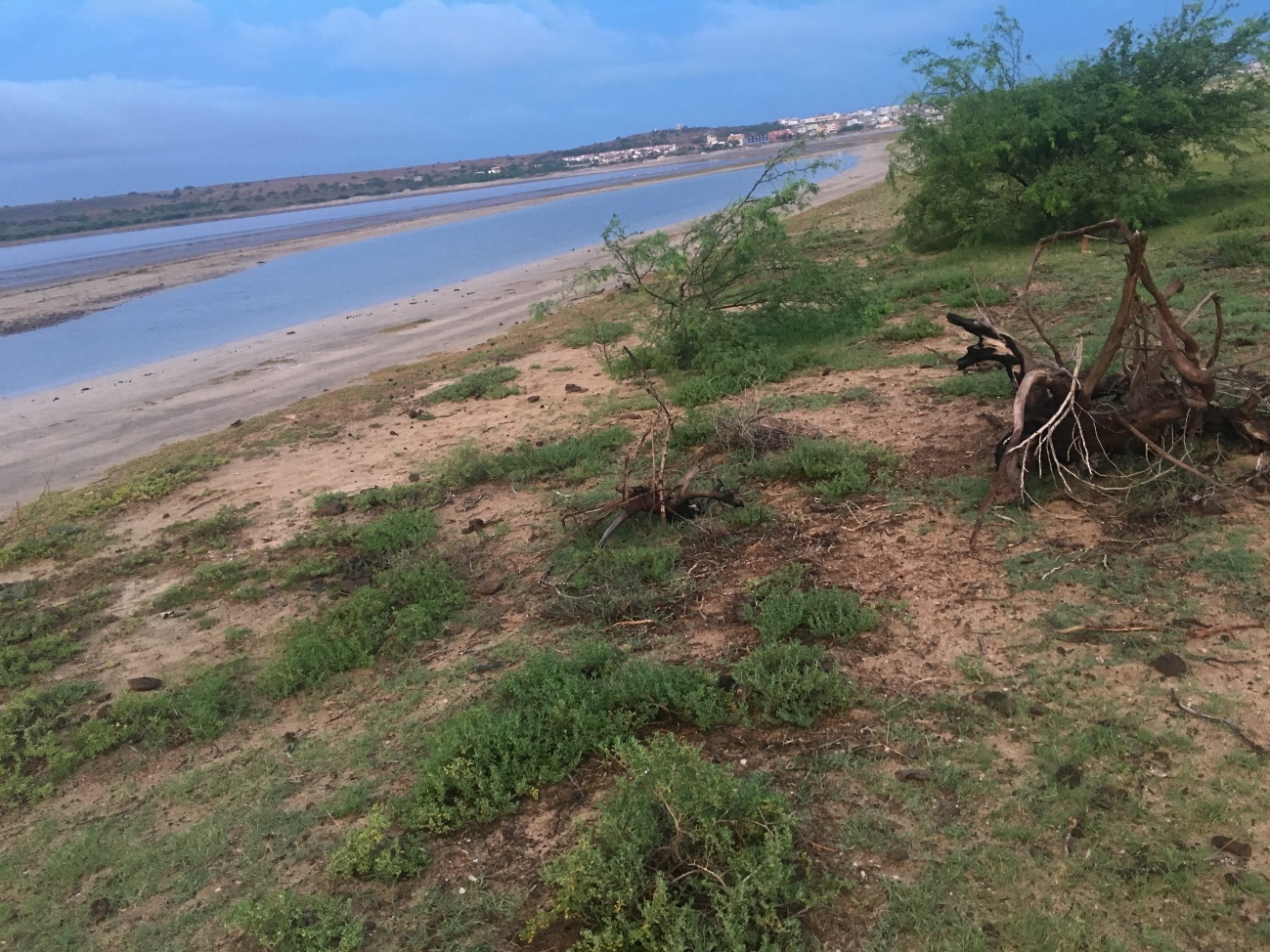


Grassland habitat: sandy area covered with small shrubs and bushes.


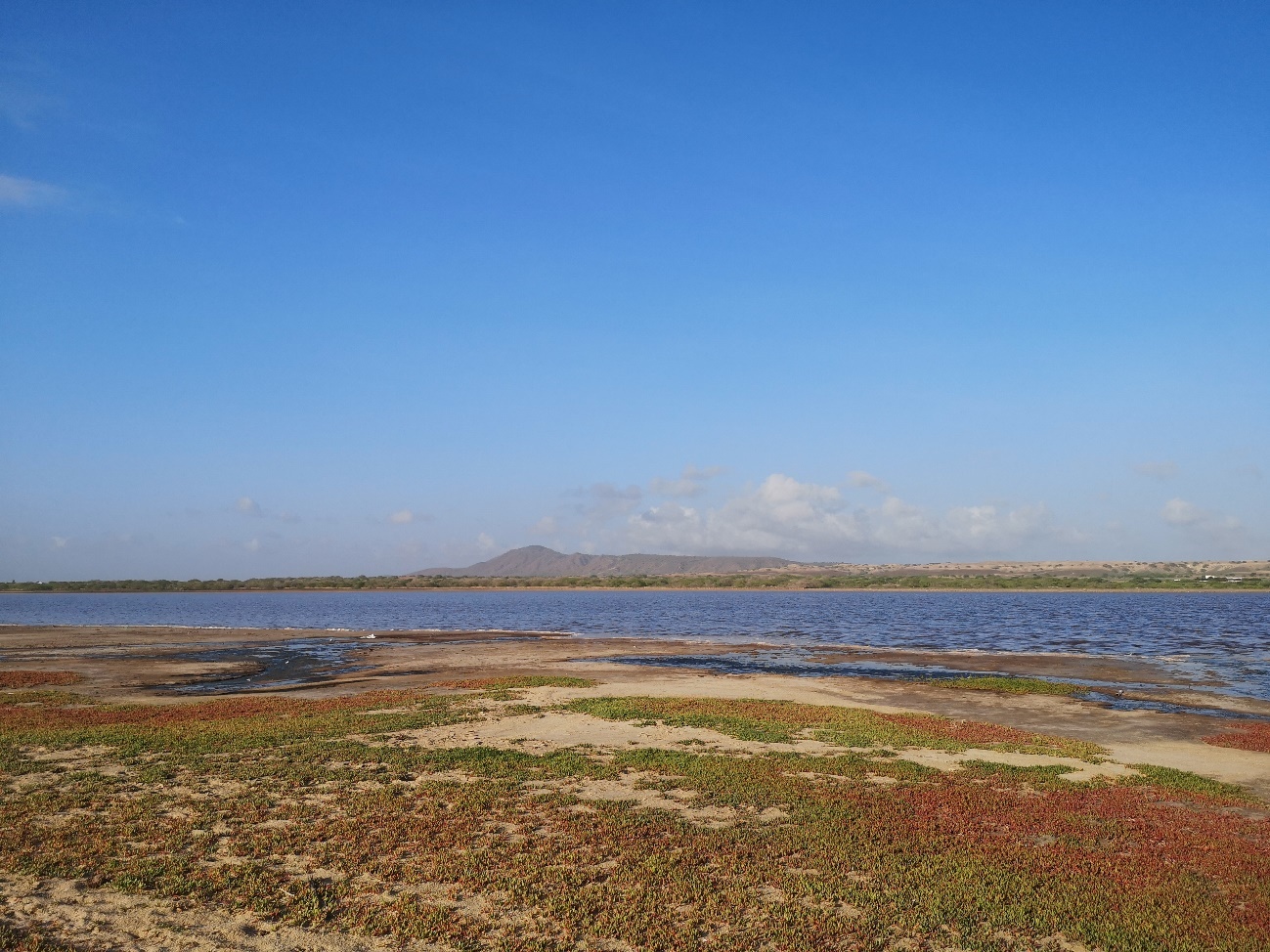


Saltmarsh habitat: sandy stretch heavily colonized by *Sesuvium portulacastrum* plants.


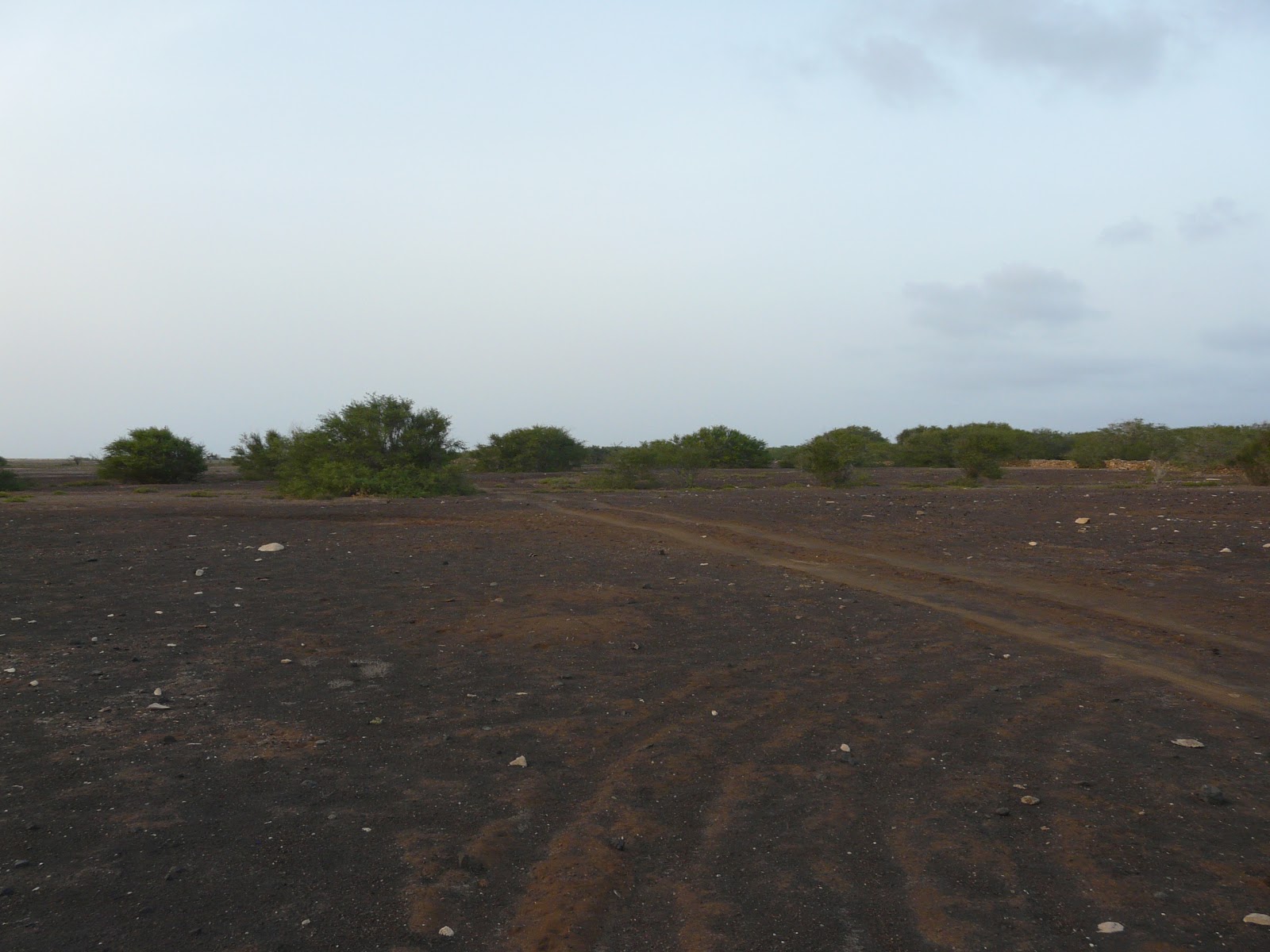


Semidesert habitat: sparsely vegetated habitat made up of mud and volcanic rocks.


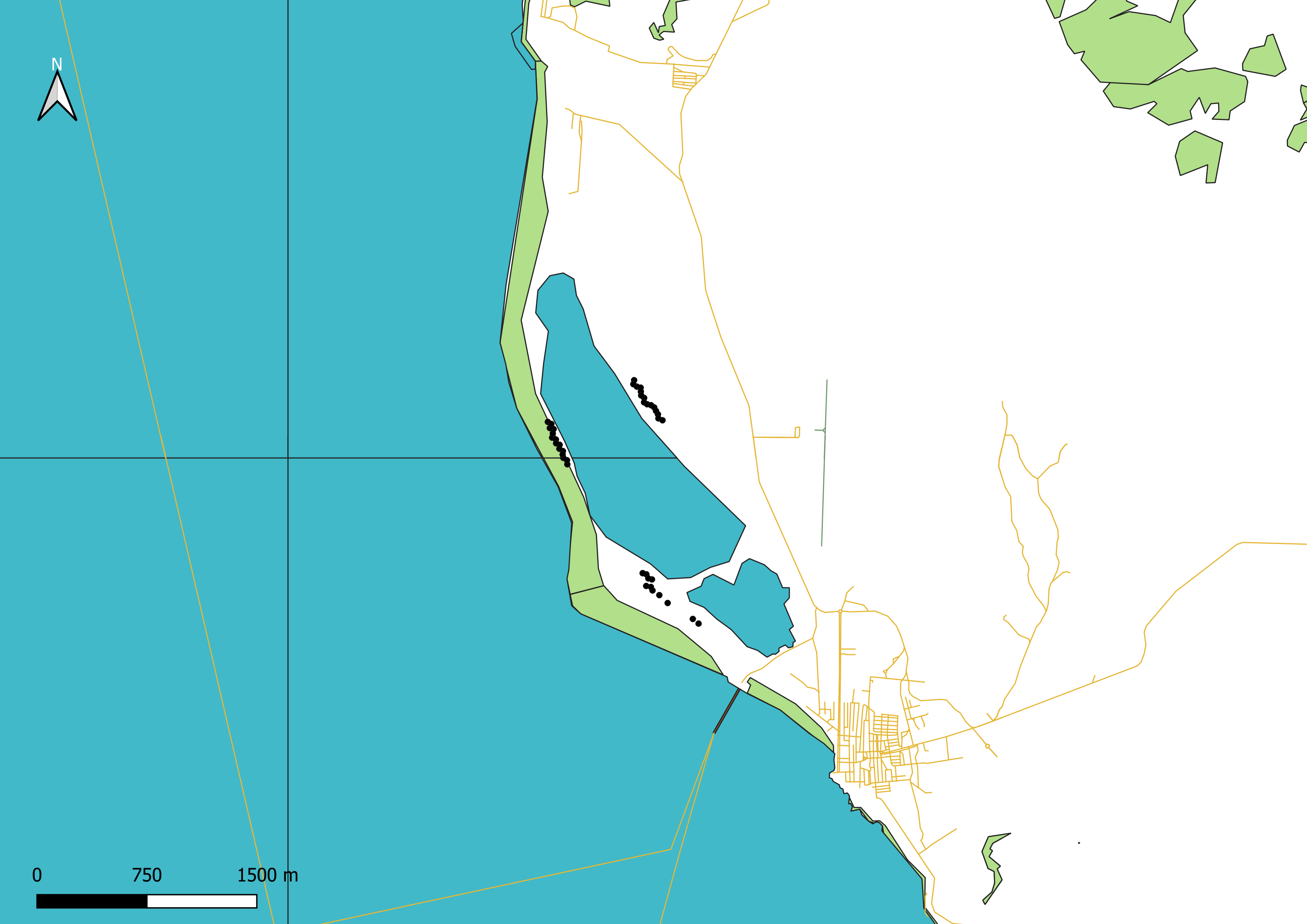


Map showing nest distributions for Trial 1 in the Salina do Porto Inglês in Maio, Cape Verde (N = 45 nests) with 15 nests in each of the three different habitats. This map was created using purchased vector tiles of Cape Verde from © MapTiler © OpenStreetMap contributors and then manipulated in QGIS.


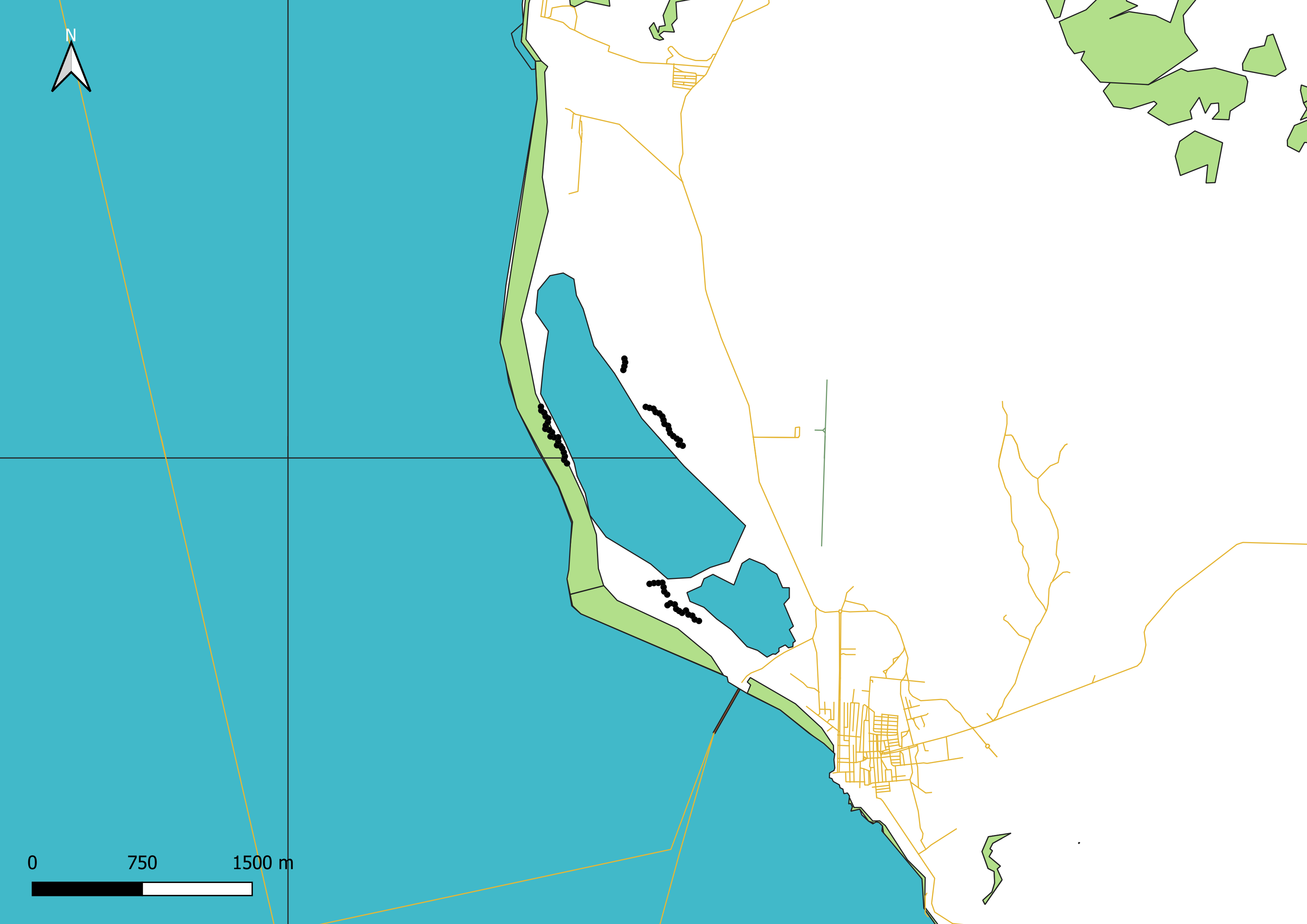


Map showing nest distributions for Trial 2 in the Salina do Porto Inglês in Maio, Cape Verde (N = 63 nests) with 21 nests in each of the three different habitats. This map was created using purchased vector tiles of Cape Verde from © MapTiler © OpenStreetMap contributors and then manipulated in QGIS.
