# Supplementary material for: Incubating parents serve as visual cues to predators in Kentish plover (Charadrius alexandrinus)
Source: PLoS One. 2020 Jul 29;15(7):e0236489. doi: 10.1371/journal.pone.0236489 (PMC7390395; doi:10.1371/journal.pone.0236489)
Supplement: S2 Appendix — (DOCX) [file pone.0236489.s002.docx]

**S2 Appendix**
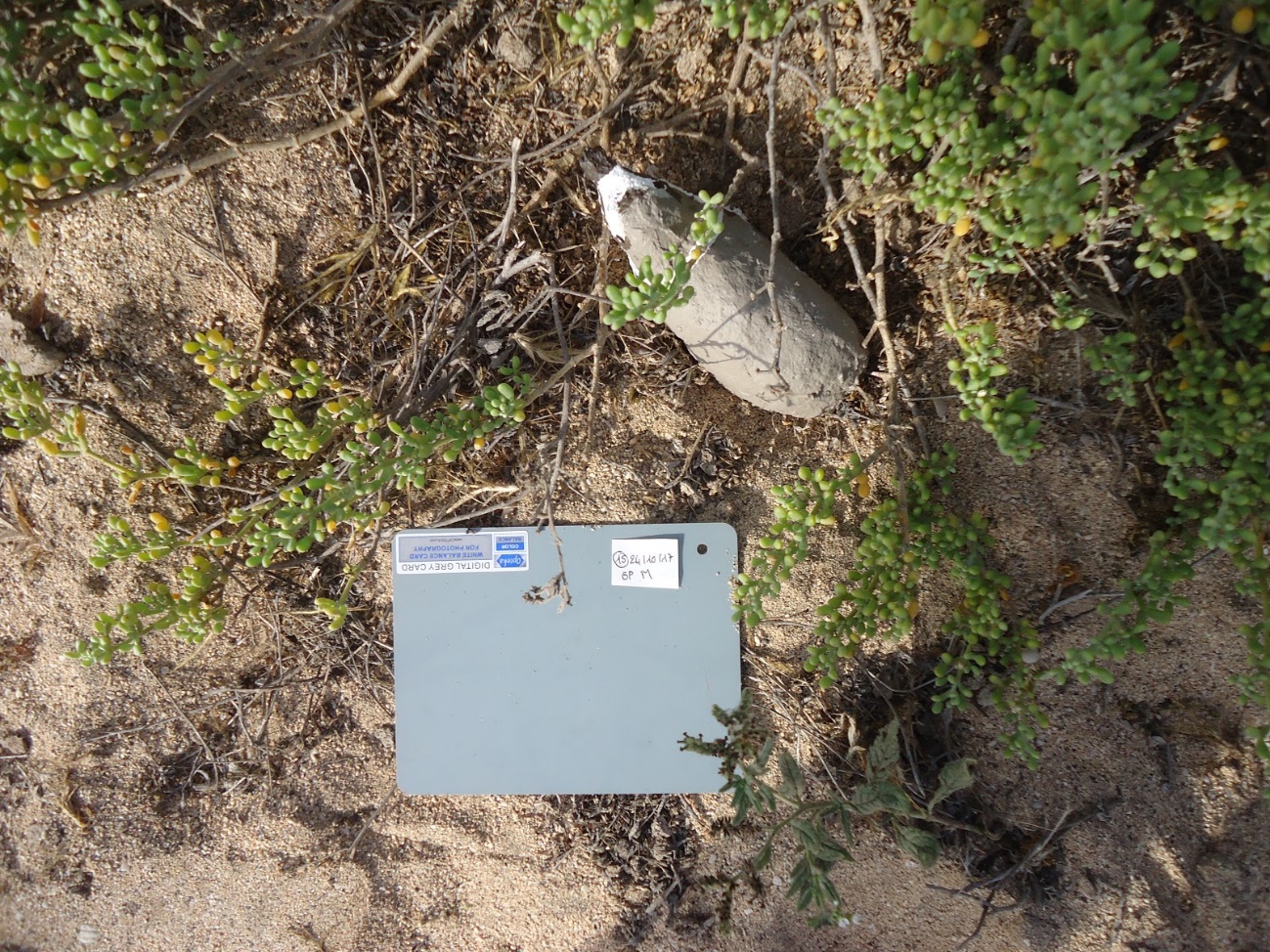


Nest example: Male decoy, Grassland (Trial 2).
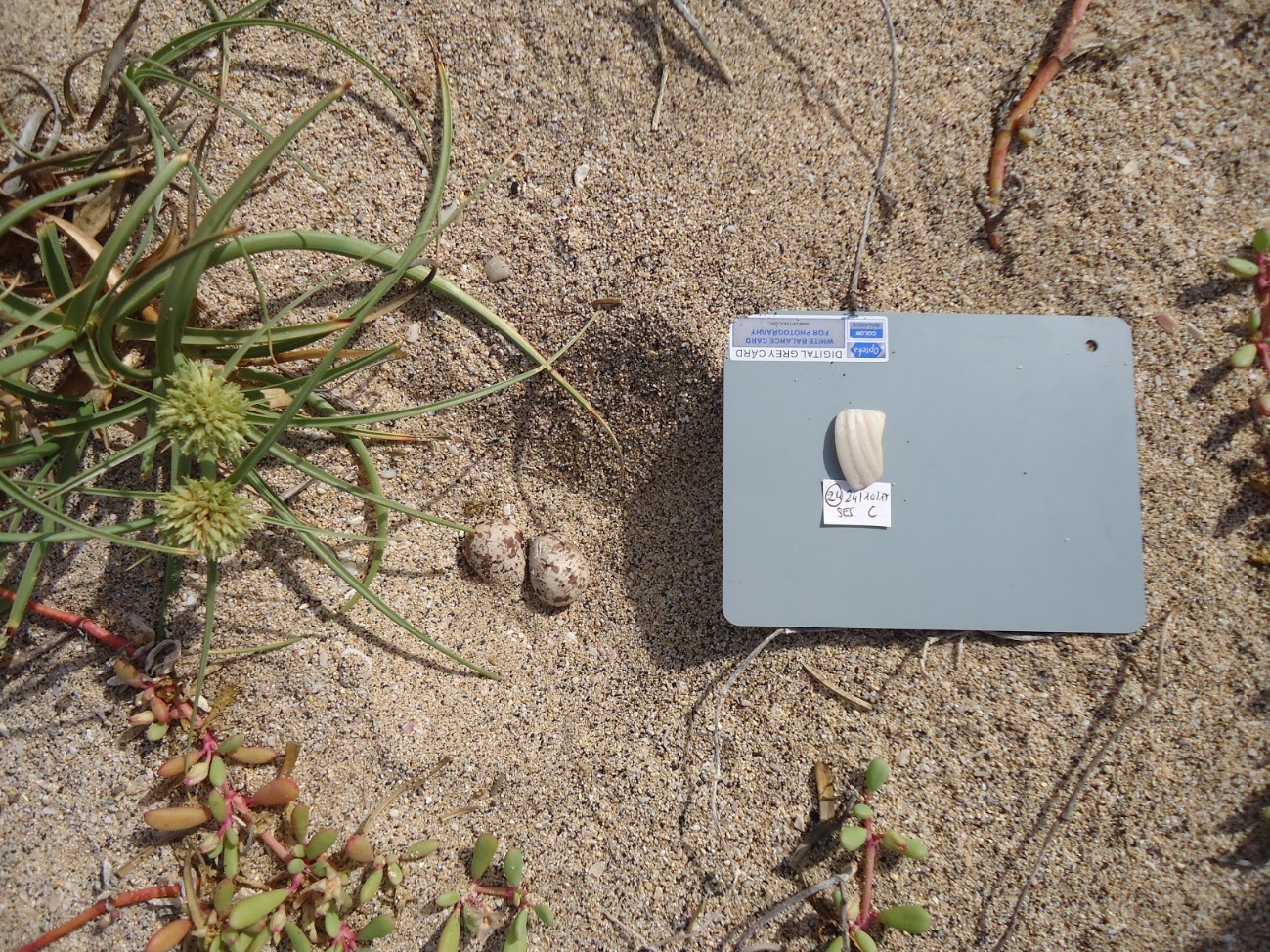


Nest Example: Control, Saltmarsh (Trial 2).


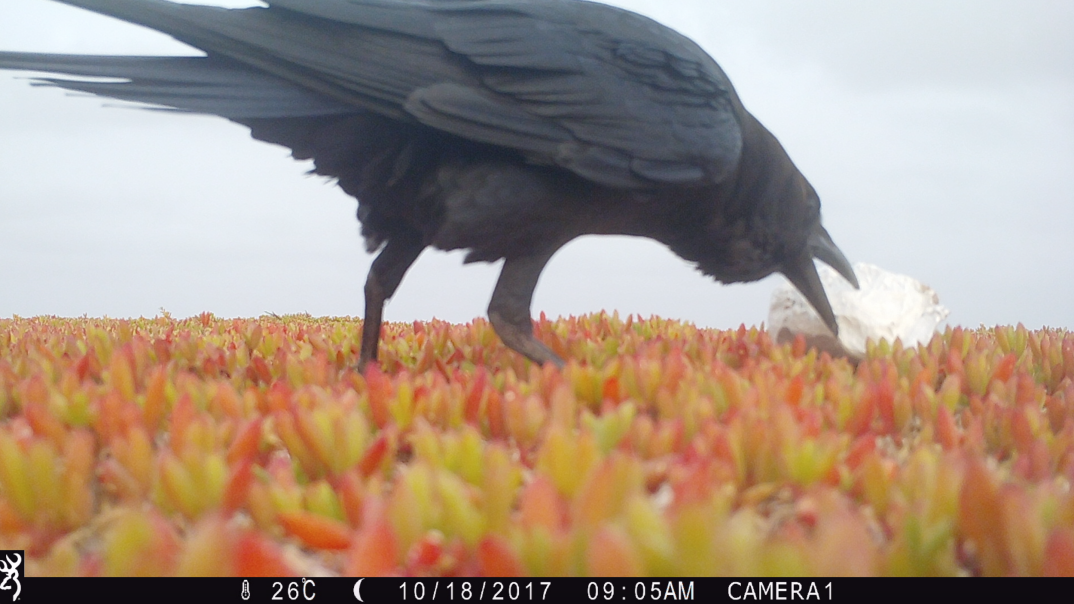


Brown-necked raven predating male plover decoy. Nest 21, Trial 1.
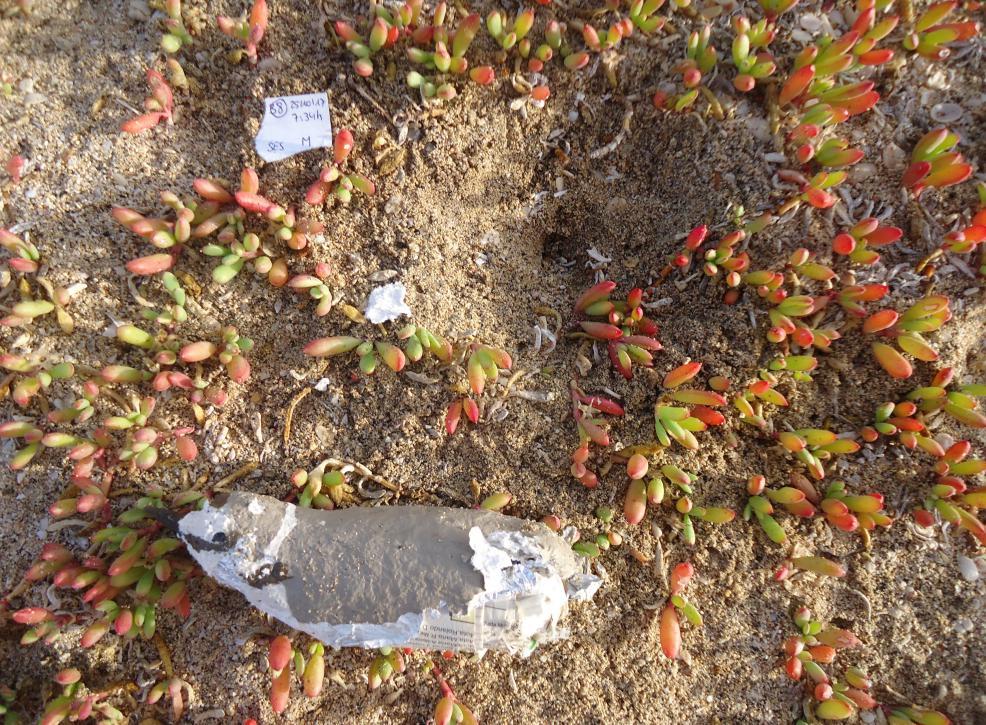


Predated artificial nest with male decoy. Nest 38, Trial 2.
