# Supplementary material for: Incubating parents serve as visual cues to predators in Kentish plover (Charadrius alexandrinus)
Source: PLoS One. 2020 Jul 29;15(7):e0236489. doi: 10.1371/journal.pone.0236489 (PMC7390395; doi:10.1371/journal.pone.0236489)
Supplement: S3 Appendix — (DOCX) [file pone.0236489.s003.docx]

**S3 Appendix**

**Table A**. **Summary of nest fates.** Number of nests that survived and that got predated in the three different habitats (N = 107). Male and female grouped as “Adult”.

|  | Grassland | | Saltmarsh | | Semidesert | |
| --- | --- | --- | --- | --- | --- | --- |
|  | Adult | Control | Adult | Control | Adult | Control |
| Survived | 1 | 9 | 0 | 0 | 0 | 6 |
| Predated | 23 | 3 | 24 | 12 | 24 | 5 |

**Table B**. **Suspected predators in the three habitats and frequency of nest predation.** Different types of suspected predators, number of predated nests and frequency of predation in the different habitats (N = 107).

| **Suspected predator** | **Habitat** | **Number of nests** | **% of nests** |
| --- | --- | --- | --- |
| ***Corvus ruficollis*** | Grassland  Saltmarsh  Semidesert | 18  15  19 | 48.60 |
| ***Ocypodinae cursor*** | Grassland  Saltmarsh  Semidesert | 4  12  7 | 21.50 |
| ***Corvus ruficollis/ Ocypodinae cursor*** | Grassland  Saltmarsh  Semidesert | 2  10  0 | 11.21 |
| **Unknown** | Grassland  Saltmarsh  Semidesert | 1  0  3 | 3.74 |
| **Survived** | Grassland  Saltmarsh  Semidesert | 10  0  6 | 14.95 |

|  | **HR** | **95% CI** | **Sig.** |
| --- | --- | --- | --- |
| **Treatment** |  |  |  |
| **Control** | 1 | . | . |
| **Male decoy** | 3.16 | 1.72 - 5.81 | < 0.001 |
| **Female decoy** | 3.43 | 1.81 - 6.48 | < 0.001 |
| **Habitat** |  |  |  |
| **Grassland** | 1 | . | . |
| **Semidesert** | 3.31 | 1.84 - 5.95 | < 0.001 |
| **Saltmarsh** | 2.79 | 1.63 - 4.78 | < 0.001 |
| **Trial** |  |  |  |
| **1-2** | 1.02 | 0.66 - 1.56 | 0.94 |

**Table C**. **Details of the full Cox hazard ratio model.** Nest survival in relation to treatment (male decoy, female decoy and control), habitat (Grassland, Saltmarsh and Semidesert) and trial. Cox hazard ratio model (N = 107 nests and number of events = 91, CI = confidence intervals, HR = Hazard ratio).
